# Estimation and determinants of direct medical costs of ischaemic heart disease, stroke and hypertensive heart disease: evidence from two major hospitals in Cameroon

**DOI:** 10.1186/s12913-021-06146-4

**Published:** 2021-02-12

**Authors:** Leopold Ndemnge Aminde, Anastase Dzudie, Yacouba N. Mapoure, Jacques Cabral Tantchou, J. Lennert Veerman

**Affiliations:** 1 School of Medicine, Griffith University, Gold Coast, Australia; 2 Clinical Research Education, Networking & Consultancy (CRENC), Douala, Cameroon; 3 Department of Internal Medicine, Douala General Hospital, Douala, Cameroon; 4 Faculty of Medicine, University of Yaounde 1, Yaounde, Cameroon; 5 Faculty of Medicine & Pharmaceutical Sciences, University of Douala, Douala, Cameroon; 6 Shisong Cardiac Centre, Kumbo, Cameroon

**Keywords:** Cost, Cardiovascular disease, Stroke, Cameroon

## Abstract

**Background:**

Cardiovascular disease (CVD) is the largest contributor to the non-communicable diseases (NCD) burden in Cameroon, but data on its economic burden is lacking.

**Methods:**

A prevalence-based cost-of-illness study was conducted from a healthcare provider perspective and enrolled patients with ischaemic heart disease (IHD), ischaemic stroke, haemorrhagic stroke and hypertensive heart disease (HHD) from two major hospitals between 2013 and 2017. Determinants of cost were explored using multivariate generalized linear models.

**Results:**

Overall, data from 850 patients: IHD (*n* = 92, 10.8%), ischaemic stroke (*n* = 317, 37.3%), haemorrhagic stroke (*n* = 193, 22.7%) and HHD (*n* = 248, 29.2%) were analysed. The total cost for these CVDs was XAF 676,694,000 (~US$ 1,224,918). The average annual direct medical costs of care per patient were XAF 1,395,200 (US$ 2400) for IHD, XAF 932,700 (US$ 1600) for ischaemic stroke, XAF 815,400 (US$ 1400) for haemorrhagic stroke, and XAF 384,300 (US$ 700) for HHD. In the fully adjusted models, apart from history of CVD event (β = − 0.429; 95% confidence interval − 0.705, − 0.153) that predicted lower costs in patients with IHD, having of diabetes mellitus predicted higher costs in patients with IHD (β = 0.435; 0.098, 0.772), ischaemic stroke (β = 0.188; 0.052, 0.324) and HHD (β = 0.229; 0.080, 0.378).

**Conclusions:**

This study reveals substantial economic burden due to CVD in Cameroon. Diabetes mellitus was a consistent driver of elevated costs across the CVDs. There is urgent need to invest in cost-effective primary prevention strategies in order to reduce the incidence of CVD and consequent economic burden on a health system already laden with the impact of communicable diseases.

**Supplementary Information:**

The online version contains supplementary material available at 10.1186/s12913-021-06146-4.

## Background

One in three adult Cameroonians have an elevated blood pressure [1], a key driver of the cardiovascular disease (CVD) burden, with main consequences being cerebrovascular accidents, hypertensive heart disease (HHD), ischemic heart disease (IHD), heart failure and chronic kidney disease [2]. Stroke, IHD and HHD were the major causes of cardiovascular death in Cameroon, and together accounted for 10% of total deaths in the country, and 24% for those aged 70 years and more [3]. CVDs are a major cause of hospitalizations globally, with heart failure responsible for about 3 to 7% of hospitalizations in general medical wards [4] and stroke admission rates increasing over five-fold between 1999 and 2012 in Cameroon [5]. These high hospitalization rates likely translate to increased healthcare costs that lead to grim economic consequences on the country, and for a setting without universal health coverage (UHC), this has significant bearing on the affected populations.

A global systematic review assessing the impact of non-communicable diseases (NCD) on healthcare spending and national incomes showed, that compared to other NCDs, CVD had the highest health expenditure, accounting for 12.0 to 16.5% of total healthcare expenses [6]. In a study assessing the cost and affordability of NCD screening, diagnosis and treatment in private and public sectors, stroke admissions were the most costly, ranging from US$ 1874 in public facilities to US$ 16,711 in private hospitals [7]. Schlatter et al. estimated the direct medical costs of IHD at a teaching hospital in Brazil and found that the mean annual cost of outpatient treatment was US$ 1521 per patient while the mean cost per hospitalization was US$ 1976 [8]. Gheorghe et al. [9], in a recent review of evidence on the economic burden of CVDs in low-income and middle-income countries (LMICs) found that the cost of treating hypertension per month was ~ US$22, while mean costs for coronary heart disease and stroke treatment ranged between US$ 300 to US$1000. In addition, the cost of treating each episode of coronary heart disease and stroke in most studies was over US$5000. Authors reported substantial variability across studies, potentially explained by differences in costing perspectives, and CVD costs were several multiples of health expenditure per capita in the majority of LMICs [9].

In Africa, very few studies have investigated the cost of CVD. In a hospital-based study from Nigeria evaluating the economic burden of heart failure, authors found that the mean total cost of care for the heart failure cohort was US$ 2128 per patient per year and payments were mostly out-of-pocket [10]. In Cameroon, Mapoure and colleagues assessed the healthcare costs of stroke during a year and found a mean cost of XAF 802,355 (~US$ 1458) per patient [11]. The study had a relatively small sample and was conducted more than 6 years ago, and so their findings may not represent the current situation. IHD and HHD are important forms of heart disease in Cameroon, found in 10 and 43% respectively of patients undergoing echocardiography [12], however, our search found no studies assessing their economic burden in Cameroon.

Data on the cost of illnesses is important to inform policy makers on the economic burden of disease and to guide decision-making for resource allocation in prevention and treatment. Given the large burden of CVD and existing gaps in the literature for Cameroon, we sought in this study to estimate the annual direct medical costs of major CVDs (IHD, stroke and HHD), and to investigate the determinants of costs in Cameroon.

## Methods

### Study design and setting

A prevalence-based cost-of-illness study was conducted in two major hospitals in Cameroon, the Douala General Hospital (DGH) and the Shisong Cardiac Centre (SCC). The DGH is located in the city of Douala, the economic capital of Cameroon, which is about 210 km west of Yaounde, the capital city. This is one of the largest referral hospitals in the country offering specialist cardiovascular care. Some diagnostic and interventional procedures conducted there include electrocardiography (ECG), echocardiography, ambulatory blood pressure monitoring, cardiac pacing, and a specialised vascular intensive care unit. It is a reputed referral and teaching hospital not only in Cameroon but also in central Africa. The SCC is located in Kumbo, a town in the Northwest region of Cameroon, which is about 459 km northwest of the capital Yaounde. It is the leading interventional cardiology centre in central Africa [13] with services to treat basic and complex cardiovascular conditions. Several diagnostic and interventional procedures are carried out there including electrophysiology, ECG, echocardiography, cardiac pacing, implantation of defibrillators and cardiac catheterizations and angiographies. These hospitals are teaching hospitals (tertiary care centres) that are among the few in the country providing comprehensive specialized cardiovascular care to patients. Though 50–70% of the patients attending are from the same region as the facilities, they receive patients from all ten regions of Cameroon, including some from neighbouring central African countries (see Supplementary file Table 1 for patient distribution).

### Study participants

**Fig. 1:**
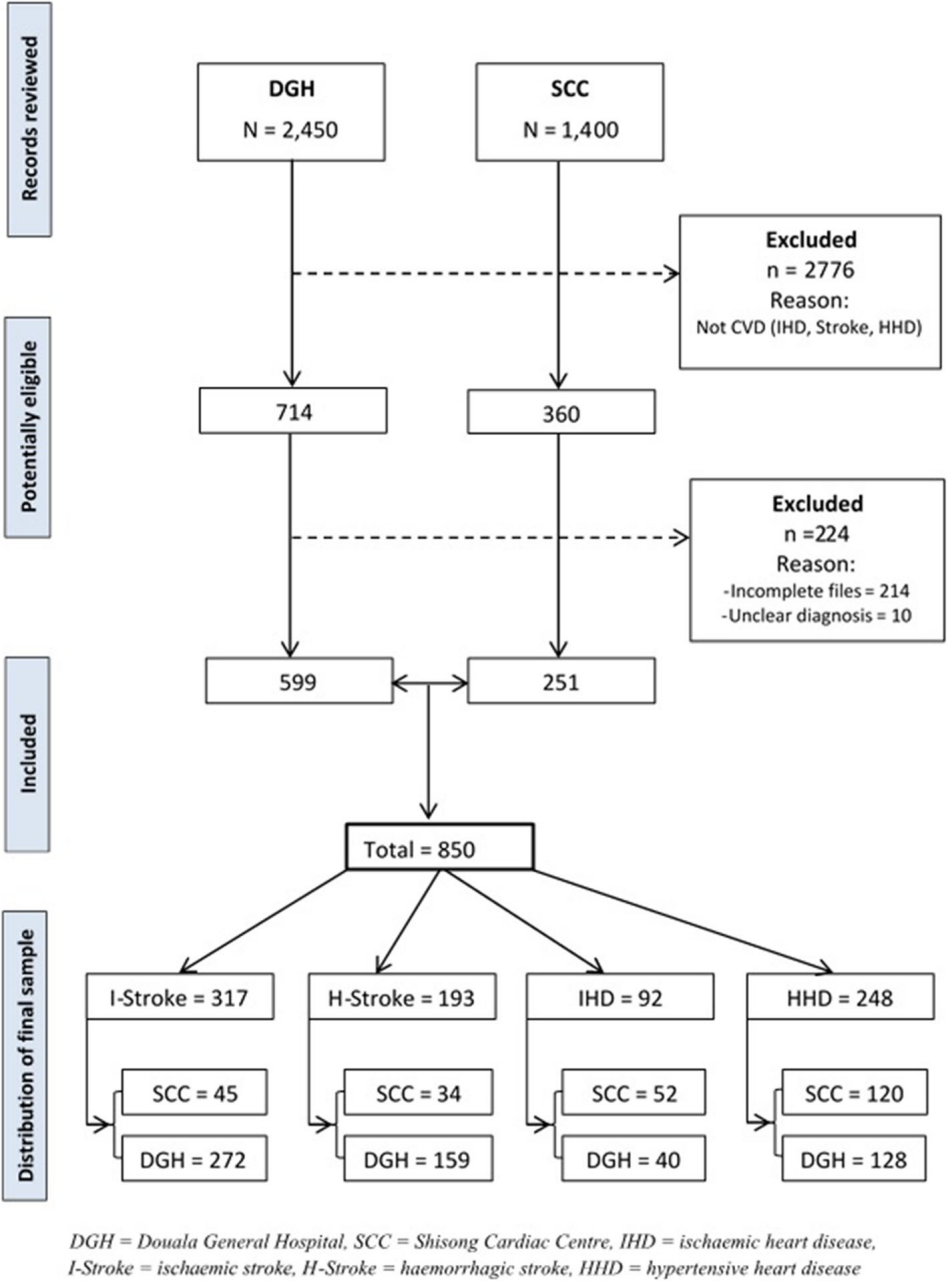
Flow chart showing selection process leading up to final study sample

We consecutively reviewed medical records of patients seen in the Cardiology and Neurology units from January 2013 to December 2017. Patients were included if they were out- or in-patient managed for hypertensive heart disease, stroke or ischemic heart disease as defined by the International Classification of Disease (ICD) 10, chapter IX codes. Specifically, this included: I11.0 and I11.9 for hypertensive heart disease; I20 – I25 for ischaemic heart disease; I61.0 to I61.9 for haemorrhagic stroke and I63.0 to I63.9 for ischaemic stroke [14]. All diagnoses were confirmed by a Consultant Cardiologist or Consultant Neurologist where appropriate. Records of patients without a clear illness definition or diagnosis, and those with largely incomplete (> 50%) data for cost estimation were excluded. For this study, we defined patients as *acute* if it was their first ever CVD event, and *prevalent* if they had a documented history of prior CVD event. Figure 1 shows the derivation of the final study sample.

### Costing perspective and approach

A healthcare system perspective [15] was used and only direct medical costs were assessed. Indirect costs as productivity loss and intangible costs were not included. A bottom-up (micro-costing) approach was used [16], in which patient-specific data were tracked for a year following initial contact based on their use of hospital services and valued. A period of 1 year was used as the aim of this study was to estimate the annual cost per patient of treating the respective conditions. The bottom-up approach was likely to provide comparatively more precision in cost estimates than macro-costing [15, 17].

### Variables and data collection

A predefined structured case report form (CRF) was used to collect data on patient characteristics and the various types and quantities of resource use from included medical records. For *patient characteristics:* sociodemographic data (age, sex, educational level, and marital status), cardiovascular risk factors (hypertension, diabetes, smoking, dyslipidaemia, atrial fibrillation, family, and previous personal history of CVD) were obtained. The latter were physician-diagnosed and reported in patient files.

### Valuation of resources

For type and quantification of resource use, the following was obtained: data on outpatient consultation (doctor and nurse) visits, laboratory tests, imaging and diagnostic tests/procedures and medications prescribed (angiotensin converting enzyme inhibitors, angiotensin receptor blockers, calcium channel blockers, beta blockers, diuretics, cardiotonic, antiarrhythmics, statins, antiplatelets, anticoagulants) were collected. For inpatient care, data on the following variables were collected; hospitalization days, intensive care unit (ICU) days, physiotherapy sessions, diagnostic/therapeutic procedures (pacemaker implantation, cardiac catheterization, and angioplasty). Post discharge, the number of follow-up/ consultations visits together with laboratory tests, any imaging and medications prescribed were also quantified.

Given it was anticipated that both hospitals most likely had different cost profiles, unit costs were obtained for each of the above resources consumed from the finance departments of both hospitals. Price lists for medications were also obtained from the respective hospital pharmacies. Unit costs for drugs that were absent from the hospital pharmacy were obtained from external pharmacies in town with a focus on generic drugs, and the lowest available cost for non-generic drugs to maintain consistency. These unit costs were then multiplied by the frequency of use/consumption of each of the resources to obtain a total cost per patient per year. Costs are expressed in local Cameroon currency (Central African CFA francs (XAF)) and converted to 2017 United States Dollars (US$) for comparative purposes (US$ 1 = XAF 552.44) [18].

### Ethical considerations

The ethical review boards of the two hospitals provided approval (Approval number: 051/DM/LHN/KC/07/2018 (DGH) and 7/08/2018 (SCC)) for this study. Patient data were anonymously reported and given this study did not involve interaction with human participants, no informed consent was required.

### Data management and statistical analysis

All data collected in the CRFs were entered to Microsoft Excel spreadsheet and randomly double-checked for accuracy and consistency. Data were then cleaned and exported to STATA 15 software (Stata Corp, College Station, TX, USA) for statistical analysis. The Kolmogorov-Smirnov test was used to check for normality of continuous data. Categorical variables were summarized as counts and percentages, while means with standard deviations (SD) (or medians with 25th and 75th percentile) were used for continuous variables. For total cost estimates, we report both mean and medians for central tendency, and SD with 25th and 75th percentiles for spread, to give a comprehensive sense of the distribution of cost data. Group comparisons were done using chi-squared tests for categorical variables. For continuous variables, parametric (t-tests/ANOVA) or non-parametric (Man-Whitney/ Kruskal Wallis) tests were used where appropriate. Healthcare costs generally have a positively skewed distribution and are non-negative, as was observed in this study. Thus, to investigate determinants of total annual costs (dependent variable) per patient for each of the CVDs, the Generalised Linear Models (GLM) with gamma family distribution and log link was used to model potential predictors. The GLM with gamma distribution has been shown to closely predict mean costs (and hence total costs) [19]. More so, the log link has the advantage of guaranteeing non-negative outcomes and maintains the original scale of the data as opposed to log transformation. Independent variables modelled included sociodemographic characteristics such as age, sex, and clinical characteristics such as hypertension, diabetes, atrial fibrillation. Univariate models were built, followed by age and sex-adjusted models (Model 1). Multivariate models (Model 2) were subsequently built for each CVD using significant variables from the basic models, and potential confounders/ predictor variables from the literature [20]. Variables adjusted for in the multivariate model (Model 2) included age, sex, hypertension, diabetes mellitus, atrial fibrillation, history of CVD event and hospital attended. A complete case analysis was performed and a *p*-value < 0.05 was considered statistically significant.

## Results

### General characteristics

A total of 850 adult patients with CVD were included. Of these, 317 (37.3%) had ischaemic stroke (mean age: 65.1 ± 12.8, male: 47.9%), 193 (22.7%) had haemorrhagic stroke (mean age: 58.8 ± 12.9, male: 51.3%), 92 (10.8%) with IHD (mean age: 57.7 ± 12.2, male: 59.8%) and 248 (29.2%) with HHD (mean age: 61.9 ± 13.5, male: 50.8%). Table 1 illustrates the patient sociodemographic and clinical characteristics. The patient mix from both hospitals included people from all ten regions of the country with majority residing in the regions wherein the hospitals are located (Supplementary file, Table 1). Prevalent hypertension ranged from 70.7% in those with IHD to 100% in those with HHD. For diabetes, the overall prevalence was 25.8%, and was highest among patients with ischaemic stroke (31.2%), *p* = 0.031. The overall prevalence of atrial fibrillation was 9.3%, but this was most common in patients with ischaemic stroke (13.6%) and those with HHD (11%), *p* < 0.001. The average length of hospital stay varied by disease; 5.0 days, 10.3 days, 13.4 days and 6.6 days for IHD, ischaemic stroke, haemorrhagic stroke and HHD, respectively.

**Table 1:** Sociodemographic and clinical characteristics of overall study sample

Characteristics	IHD (*n* = 92, 10.8%)	I-stroke (*n* = 317, 37.3%)	H-stroke (*n* = 193, 22.7%)	HHD (*n* = 248, 29.2%)	Total (*n* = 850)	***p***-value
***Socio-demographic***						
**Age, years,** mean ± SD (min – max)	57.7 ± 12.2 (32–85)	65.1 ± 12.8 (31–95)	58.8 ± 12.9 (26–95)	61.9 ± 13.5 (26–92)	61.8 ± 13.3 (26–95)	0.001*
**Sex, male**	55 (59.8)	152 (47.9)	99 (51.3)	126 (50.8)	432 (50.8)	0.259
**Marital status**						< 0.001
Single	4 (10.3)	14 (5.1)	12 (7.5)	12 (9.4)	42 (7.0)	
Married	21 (53.8)	188 (69.1)	117 (73.6)	92 (71.9)	418 (69.9)	
Divorced	6 (15.4)	4 (1.5)	3 (1.9)	10 (7.8)	23 (3.8)	
Widow (er)	8 (20.5)	66 (24.3)	27 (17.0)	14 (10.9)	115 (19.2)	
**Level of Education**						< 0.001
None/Primary	5 (12.5)	32 (25.6)	19 (20.0)	21 (16.7)	77 (20.0)	
Secondary	15 (37.5)	47 (37.6)	38 (40.0)	52 (41.3)	152 (39.4)	
High school	11 (27.5)	5 (4.0)	8 (8.4)	24 (19.0)	48 (12.4)	
University	9 (22.5)	41 (32.8)	30 (31.6)	29 (23.0)	109 (28.2)	
**Study hospital**						< 0.001
DGH	40 (43.5)	272 (85.8)	159 (82.4)	128 (51.6)	599 (70.5)	
SCC	52 (56.5)	45 (14.2)	34 (17.6)	120 (48.4)	251 (29.5)	
***Clinical/risk factors***						
**Hypertension**	65 (70.7)	246 (77.6)	175 (90.7)	248 (100)	734 (86.2)	< 0.001
**Diabetes**	17 (20.5)	99 (31.2)	48 (24.9)	48 (21.1)	212 (25.8)	0.031
**Smoking**	5 (6.2)	38 (12.0)	23 (11.9)	15 (7.0)	81 (10.1)	< 0.001
**Dyslipidaemia**	4 (4.4)	25 (7.9)	7 (3.6)	10 (4.9)	46 (5.7)	0.113
**Atrial fibrillation**	2 (2.5)	43 (13.6)	6 (3.1)	24 (11.0)	75 (9.3)	0.001
**Past history of CVD**	34 (40.0)	93 (29.3)	46 (23.8)	107 (46.3)	280 (33.9)	< 0.001
**Family history of CVD**	7 (8.0)	106 (33.4)	54 (28.0)	33 (14.0)	200 (24.0)	< 0.001
**Physical inactivity**	16 (17.8)	155 (48.9)	75 (38.9)	84 (34.3)	330 (39.1)	0.001
**Overweight/obesity**	11 (13.4)	109 (34.4)	47 (24.4)	38 (16.7)	205 (25.0)	0.001
**Length of hospital stay**						< 0.001^#^
Mean ± SD	5.0 ± 3.3	10.3 ± 9.7	13.4 ± 11.7	6.6 ± 4.4	9.2 ± 8.9	
Median (25th – 75th %)	5 (3–6)	8.0 (5–12)	12 (6–17)	6 (4–8)	7 (4–12)	

*CVD* Cardiovascular disease, *IHD* Ischaemic heart disease, *I-stroke* Ischaemic stroke, *H-stroke* Haemorrhagic stroke, *HHD* Hypertensive heart disease, *DGH* Douala General Hospital, *SCC* Shisong Cardiac Centre, *SD* Standard deviation, *min* Minimum, *max* Maximum, *Differences in post hoc analysis were all significant except for IHD vs. H-Stroke, IHD vs. HHD and H-stroke vs. HHD that were not significant, ^#^Differences between groups in post hoc analysis were all significant except for IHD vs. HHD with no difference

### Total direct annual medical costs

The total cost for all four CVDs combined was XAF 676,694,300 (~US$ 1,224,918), mostly driven by stroke costs: XAF 295,662,600 (~ US$ 535,194); 43.7% and XAF 157,370,700 (~US$ 284,865); 23.2% for ischaemic and haemorrhagic stroke respectively. The annual mean total cost was XAF 1,395,200 (~US$ 2525) for IHD, XAF 932,700 (~US$ 1688) for ischaemic stroke, XAF 815,400 (~US$ 1476) for haemorrhagic stroke and XAF 384,300 (~US$ 695) for HHD (Table 2). The annual mean costs were higher at the DGH compared to the SCC across all CVDs except for IHD where the average cost was much higher at the SCC (XAF 2,097,300, ~US$ 3796 vs. XAF 482,400, ~US$ 873) (Supplementary file, Tables 2, 3, 4, 5). Average costs of all CVDs were largely similar for people residing in same region as for those from other regions in the country (Supplementary file, Tables 6 and 7).

**Table 2:** Direct medical costs for patients with Ischaemic heart disease, Ischaemic and Haemorrhagic stroke and Hypertensive heart disease stratified by resource use for the two study hospitals

	IHD *n* = 92	I-stroke *n* = 317	H-stroke *n* = 193	HHD *n* = 248	Overall *N* = 850
**Consultation**					
*Mean (SD)*	6300 (5200)	13,700 (9800)	10,400 (7300)	7400 (7400)	10,300 (8700)
	[11.4 (9.4)]	[24.8 (17.7)]	[18.8 (13.2)]	[13.4 (13.4)]	[18.6 (15.7)]
*Median (25th – 75th percentile)*	3500 (3500 – 7000)	7000 (7000 – 21,000)	7000 (7000 – 14,000)	7000 (3500 – 7000)	7000 (3500 – 14,000)
	[6.3 (6.3–12.7)]	[12.7 (12.7–38.0)]	[12.7 (12.7–25.3)]	[12.7 (6.3–12.7)]	[12.7 (6.3–25.3)]
*Sum (% of total costs)*	577,500 (0.5)	4,336,500 (1.5)	2,009,000 (1.3)	1,841,000 (1.9)	8,764,000 (1.2)
	[1045.4]	[7849.7]	[3636.6]	[3332.5]	[15,864.2]
**Laboratory tests**					
*Mean (SD)*	96,300 (58,700)	111,000 (74,300)	100,300 (80,900)	102,700 (109,600)	104,500 (86,000)
	[174.3 (106.3)]	[200.9 (134.5)]	[181.5 (146.4)]	[185.9 (198.4)]	[189.1 (155.6)]
*Median (25th – 75th percentile)*	67,700 (63,400–131,300)	103,900 (55,000–144,900)	82,100 (49,700–126,200)	58,300 (20,300 – 150,400)	85,800 (41,100 – 140,800)
	[122.5 (114.7–237.7)]	[188.0 (99.5–262.3)]	[148.6 (89.9–228.4)]	[105.5 (36.7–272.2)]	[155.3 (74.4–254.8)]
*Sum (% of total costs)*	8,856,500 (6.9)	34,646,800 (11.7)	19,258,600 (12.2)	24,742,700 (25.9)	87,504,600 (12.9)
	[16,031.6]	[62,715.9]	[34,860.9]	[44,788.0]	[158,396.5]
**Imaging & procedures**					
*Mean (SD)*	1,072,000 (1,356,000)	193,300 (115,600)	192,000 (317,700)	74,800 (124,900)	262,000 (575,600)
	[1940.4 (2454.5)]	[349.9 (209.2)]	[347.5 (575.1)]	[135.4 (226.1)]	[474.2 (1041.9)]
*Median (25th – 75th percentile)*	675,000 (61,300 – 2,700,000)	176,000 (90,000 – 266,000)	143,100 (75,000 – 200,800)	50,800 (30,000 – 71,300)	125,800 (50,800 – 229,300)
	[1221.8 (110.9–4887.4)]	[318.6 (162.9–481.5)]	[259.0 (135.7–363.4)]	[91.9 (54.3–129.1)]	[227.7 (91.9–415.1)]
*Sum (% of total costs)*	98,623,800 (76.8)	58,582,300 (19.8)	34,941,300 (22.2)	16,374,500 (17.2)	208,521,900 (30.8)
	[178,524.0]	[106,042.8]	[63,249.0]	[29,640.3]	[377,456.2]
**Medication**					
*Mean (SD)*	188,200 (191,900)	332,800 (238,900)	291,100 (221,700)	166,100 (118,500)	258,000 (213,400)
	[340.6 (347.3)]	[602.4 (432.4)]	[526.9 (401.3)]	[300.7 (214.5)]	[467.0 (386.3)]
*Median (25th – 75th percentile)*	127,900 (54,800 – 244,700)	277,300 (150,500 – 447,900)	258,500 (130,300 – 399,600)	136,800 (76,700 – 232,000)	210,600 (103,100 – 349,100)
	[231.5 (99.2–442.9)]	[501.9 (272.4–810.7)]	[467.9 (235.8–723.3)]	[247.6 (138.8–419.9)]	[381.2 (186.6–631.9)]
*Sum (% of total costs)*	17,317,800 (13.5)	103,837,600 (35.1)	51,528,300 (32.7)	41,200,700 (43.2)	213,884,400 (31.6)
	[31,347.8]	[187,961.7]	[93,274.0]	[74,579.5]	[387,163.1]
**Hospitalization**					
*Mean (SD)*	32,400 (48,700)	173,100 (212,500)	240,500 (277,500)	44,900 (61,500)	131,300 (200,900)
	[58.6 (88.2)]	[313.3 (384.6)]	[435.3 (502.3)]	[81.3 (111.3)]	[237.7 (363.6)]
*Median (25th – 75th percentile)*	8000 (4300 – 56,800)	104,100 (42,600 – 261,400)	127,800 (28,400 – 379,400)	26,700 (7000 – 71,000)	56,800 (11,000 – 161,500)
	[14.5 (7.8–102.8)]	[188.4 (77.1–473.2)]	[231.3 (51.4–686.7)]	[48.3 (12.6–128.5)]	[102.8 (20.0–292.3)]
*Sum (% of total costs)*	2,982,100 (2.3)	52,613,900 (17.8)	39,195,000 (24.9)	11,144,400 (11.8)	105,935,400 (15.7)
	[5398.0]	[95,239.1]	[70,948.9]	[20,173.0]	[191,759.1]
**Physiotherapy**					
*Mean (SD)*	–	578,400 (503,000)	401,500 (368,900)	–	531,500 (475,900)
		[1047.0 (910.5)]	[726.8 (667.7)]		[962.1 (861.4)]
*Median (25th – 75th percentile)*	–	408,500 (152,000 – 988,000)	342,000 (41,300 – 717,300)	–	389,500 (152,000 – 883,500)
		[739.4 (275.1–1788.4)]	[619.1 (74.8–1298.4)]		[705.0 (275.1–1599.3)]
*Sum (% of total costs)*	–	41,645,500 (14.1)	10,438,500 (6.7)	–	52,084,000 (7.8)
		[75,384.6]	[18,895.3]		[94,280.0]
**Total Costs**					
*Mean (SD)*	1,395,200 (1,367,000)	932,700 (675,900)	815,400 (705,600)	384,300 (300,500)	796,100 (777,600)
	[2525.5 (2474.5)]	[1688.3 (1223.5)]	[1476.0 (1277.2)]	[695.6 (543.9)]	[1441.0 (1407.6)]
*Median (25th – 75th percentile)*	773,400 (432,100 – 2,940,000)	797,000 (449,700 – 1,258,200)	611,600 (306,400 – 1,117,400)	298,100 (180,400 – 526,600)	571,500 (275,900 – 1,028,100)
	[1400.0 (782.2–5321.8)]	[1442.7 (814.0–2277.5)]	[1107.1 (554.6–2022.6)]	[539.6 (328.4–953.2)]	[1034.5 (499.4–1861.0)]
*Sum for each CVD and overall*	128,357,700	295,662,600	157,370,700	95,303,300	676,694,300
	[232,346.8]	[535,194.0]	[284,864.8]	[172,513.4]	[1,224,919.1]
*% of total CVD costs combined*	19.0	43.7	23.2	14.1	100.0

Costs are in Central African francs (XAF). For ease with comprehension and comparisons, equivalent costs expressed in US dollars are presented in square brackets. *CVD* Cardiovascular disease, *IHD* Ischaemic heart disease, *I-stroke* Ischaemic stroke, *H-stroke* Haemorrhagic stroke, *HHD* Hypertensive heart disease, *SD* Standard deviation

### Acute versus prevalent CVD costs

The mean annual cost was XAF 1,849,100 (~US$ 3347) for acute IHD patients and XAF 911,500 (~ US$ 1650) for prevalent IHD cases. For haemorrhagic stroke patients, the mean annual cost was XAF 827,400 (~US$ 1497) per acute case, while prevalent cases had a mean cost of XAF 776,900 (~US$ 1406). With respect to HHD, mean annual cost was XAF 400,500 (~US$ 725) per acute case, while the mean cost for prevalent HHD was XAF 346,600 (~US$ 627). The average annual cost per acute ischaemic stroke case was XAF 920,000 (~US$ 1665) while prevalent ischaemic stroke had a mean annual cost of XAF 960,600 (~US$ 1738). Higher costs in acute CVD cases were mostly driven by imaging and medication costs. However, for both forms of stroke, physiotherapy was a major contributor especially to the higher costs in prevalent ischaemic stroke (Table 3).

**Table 3:** Annual direct medical costs for patients with ischaemic heart disease, ischaemic stroke, haemorrhagic stroke and hypertensive heart disease by disease stage attending two hospitals in Cameroon, 2013–2017

	IHD		I-stroke		H-stroke		HHD	
	Acute (*n* = 60, 65.2%)	Prevalent (*n* = 32, 34.8%)	Acute (*n* = 224, 70.6%)	Prevalent (*n* = 93, 29.4%)	Acute (*n* = 147, 76.2%)	Prevalent (*n* = 46, 23.8%)	Acute (*n* = 115, 46.4%)	Prevalent (*n* = 133, 53.6%)
**Consultation**	5700 (4200)	6400 (5700)	13,900 (10,100)	13,100 (9100)	10,700 (7500)	9400 (6700)	7300 (7900)	7200 (7000)
	[10.3 (7.6)]	11.6 (10.3)]	[25.2 (18.3)]	[23.7 (16.5)]	[19.4 (13.6)]	[17.0 (12.1)]	[13.2 (14.3)]	[13.0 (12.6)]
**Laboratory tests**	84,600 (55,000)	104,800 (59,100)	110,800 (76,700)	111,700 (68,700)	99,900 (77,200)	101,500 (93,000)	99,300 (114,900)	93,600 (102,800)
	[153.1 (99.6)]	[189.7 (106.9)]	[200.6 (138.8)]	[211.8 (124.3)]	[180.8 (139.7)]	[183.7 (168.3)]	[179.7 (208.0)]	[169.4 (186.1)]
**Imaging and procedures**	1,526,100 (1,569,600)*	581,100 (766,100)	196,100 (119,200)	186,500 (106,700)	206,100 (355,300)	144,900 (121,400)	86,400 (172,300)	60,200 (39,400)
	[2762.5 (2841.2)]	[1051.8 (1386.7)]	[354.9 (215.8)]	[337.6 (193.1)]	[373.1 (643.1)]	[262.3 (219.7)]	[156.4 (311.9)]	[109.0 (71.3)]
**Medication**	206,700 (197,800)	181,800 (197,200)	330,700 (224,500)	338,000 (272,000)	298,000 (233,700)	270,500 (181,400)	176,000 (132,700)	158,400 (103,300)
	[374.1 (258.0)]	[329.1 (356.9)]	[598.6 (406.4)]	[611.8 (492.3)]	[539.4 (423.0)]	[489.6 (328.3)]	[318.6 (240.2)]	[286.7 (187.0)]
**Hospitalization**	26,000 (40,700)	37,500 (61,700)	164,800 (171,100)	193,100 (289,400)	259,700 (297,100)	186,700 (207,000)	46,600 (75,500)	36,200 (40,400)
	[47.0 (73.7)]	[67.9 (111.7)]	[298.3 (309.7)]	[349.5 (523.8)]	[470.1 (537.8)]	[337.9 (374.7)]	[84.3 (136.7)]	[65.5 (73.1)]
**Physiotherapy**	**–**	**–**	564,900 (519,100)	607,200 (477,000)	380,900 (348,000)	429,500 (411,400)	**–**	**–**
			[1022.5 (939.6)]	[1099.1 (863.4)]	[689.5 (629.9)]	[777.4 (744.7)]		
**Total**	1,849,100 (1,602,700)*	911,500 (708,700)	920,000 (621,500)	960,600 (794,600)	827,400 (735,900)	776,900 (604,200)	400,500 (351,500)	346,600 (241,300)
	[3347.1 (2901.1)]	[1650.0 (1282.8)]	[1665.3 (1125.0)]	[1738.8 (1438.3)]	[1497.7 (1332.0)]	[1406.3 (1093.7)]	[725.0 (636.2)]	[627.4 (436.8)]

Costs are means (standard deviations). Patients were categorized as ‘*acute’* as those who presented as a first ever CVD event, while a patient was categorized as ‘*prevalent’* if they had a documented history of prior CVD event. Costs are in Central African francs (XAF). For ease with comparisons, equivalent costs expressed in United States dollars (USD) are presented in square brackets. *CVD* Cardiovascular disease, *IHD* Ischaemic heart disease, *I-stroke* Ischaemic stroke, *H-stroke* Haemorrhagic stroke, *HHD* Hypertensive heart disease. *significant difference between acute and prevalent IHD for imaging & procedure cost category and for total costs (*p* < 0.01)

### Inpatient versus outpatient CVD costs

**Fig. 2:**
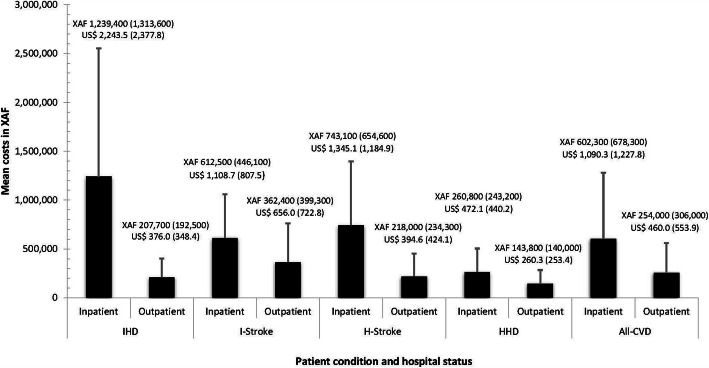
Average annual healthcare costs per patient for each CVD by patient hospital status. Costs are presented in the figure as mean (standard deviation) in central African franc (XAF) above and United States dollar (USD) below. The bars represent the mean costs while the whiskers refer to the standard deviation. IHD = Ischaemic heart disease, I-stroke = ischaemic stroke, H-stroke = haemorrhagic stroke, HHD = hypertensive heart disease

Figure 2 depicts the distribution of inpatient and outpatient costs. Overall, the mean inpatient cost for all four CVDs combined was XAF 602,300 (~US$ 1090) per patient, while mean outpatient cost was XAF 254,000 (~US$ 460). Inpatient costs were higher than outpatient costs across all four CVDs. The inpatient costs were highest for IHD (XAF 1,239,400; ~US$ 2243), followed by haemorrhagic stroke (XAF 743,100; ~US$ 1345), then ischaemic stroke (XAF 612,500; ~US$ 1108) and lastly HHD (XAF 260,800; ~US$ 472). Average outpatient cost varied from XAF 143,800 (~US$ 260) for HHD to XAF 362,400 (~US$ 656) for ischemic stroke.

### Determinants of total cost for each CVD

In the fully adjusted multivariable models, having diabetes mellitus (β = 0.435; 95% confidence interval (CI) 0.098 to 0.772) predicted increased IHD annual costs while a history of prior CVD event (β = − 0.429; − 0.705 to − 0.153) was associated with lower IHD costs. In patients with ischaemic stroke, the presence of diabetes mellitus (β = 0.188; 0.052 to 0.324) was associated with higher healthcare costs. We found no significant predictor for haemorrhagic stroke. Finally, among patients with HHD, having diabetes mellitus (β = 0.229; 0.080 to 0.378) predicted elevated annual healthcare costs, Tables 4 and 5.

**Table 4:** Predictors of annual direct medical costs for patients with ischaemic and hypertensive heart diseases attending two major hospitals in Cameroon, 2013–2017

**Ischaemic heart disease**			
**Variable**	**Univariate models** β (95% CI)	**Model 1** β (95% CI)	**Model 2** β (95% CI)
Age, *per year increase*	− 0.014 (− 0.032, 0.003)	− 0.012 (− 0.030, 0.005)	− 0.006 (− 0.017, 0.006)
Sex (*Reference: Female)*			
*Male*	0.313 (− 0.054, 0.681)	0.269 (− 0.102, 0.640)	0.025 (− 0.247, 0.297)
History of hypertension *(Reference: No)*			
*Yes*	0.319 (0.077, 0.716)*	0.352 (0.041, 0.746)*	0.070 (− 0.238, 0.378)
History of diabetes *(Reference: No)*			
*Yes*	0.223 (−0.246, 0.691)	0.370 (− 0.096, 0.836)	0.435 (0.098, 0.772)*
History of atrial fibrillation *(Reference: No)*			
*Yes*	−1.465 (−2.698, − 0.232)*	− 1.489 (− 2.716, − 0.263)*	−0.485 (− 1.361, 0.390)
History of CVD event *(Reference: No)*			
*Yes*	−0.611 (− 0.973, − 0.249)**	−0.630 (− 0.993, − 0.268)**	− 0.429 (− 0.705, − 0.153)**
Intercept			14.841 (14.125, 15.557)***
**Hypertensive heart disease**			
**Variable**	**Univariate models** β (95% CI)	**Model 1** β (95% CI)	**Model 2** β (95% CI)
Age, *per year increase*	−0.001 (− 0.007, 0.005)	− 0.001 (− 0.008, 0.005)	−0.003 (− 0.007, 0.002)
Sex (*Reference: Female)*			
*Male*	−0.047 (− 0.219, 0.126)	−0.051 (− 0.224, 0.123)	−0.012 (− 0.128, 0.105)
History of diabetes *(Reference: No)*			
*Yes*	0.387 (0.174, 0.600)***	0.389 (0.176, 0.603)***	0.229 (0.080, 0.378)**
History of atrial fibrillation *(Reference: No)*			
*Yes*	0.328 (0.040, 0.617)*	0.377 (0.075, 0.679)*	0.020 (− 0.191, 0.231)
History of CVD event *(Reference: No)*			
*Yes*	−0.175 (− 0.348, − 0.002)*	−0.173 (− 0.348, 0.001)	−0.081 (− 0.200, 0.037)
Intercept			12.318 (12.017, 12.619)***

Parameter estimates from Generalized Linear models with gamma distribution and Log-link function. Model 1: Univariate models + adjusted for age and sex, Model 2: Multivariate model adjusting for age, sex, hypertension, diabetes, atrial fibrillation & previous CVD event and hospital attended. * < 0.05, ** < 0.01, ** < 0.001

**Table 5:** Predictors of annual direct medical costs for patients with ischaemic and haemorrhagic stroke attending two major hospitals in Cameroon, 2013–2017

**Ischaemic stroke**			
**Variable**	**Univariate models** β (95% CI)	**Model 1** β (95% CI)	**Model 2** β (95% CI)
Age, *per year increase*	0.008 (0.002, 0.014)*	0.007 (0.001, 0.014)*	0.003 (−0.002, 0.008)
Sex (*Reference: Female)*			
*Male*	−0.124 (− 0.283, 0.034)	−0.095 (− 0.254, 0.064)	−0.077 (− 0.201, 0.048)
History of hypertension *(Reference: No)*			
*Yes*	−0.142 (− 0.332, 0.048)	−0.175 (− 0.364, 0.014)	−0.143 (− 0.295, 0.009)
History of diabetes *(Reference: No)*			
*Yes*	0.203 (0.033, 0.373)*	0.209 (0.040, 0.378)*	0.188 (0.052, 0.324)*
History of atrial fibrillation *(Reference: No)*			
*Yes*	0.096 (−0.136, 0.328)	0.052 (−0.179, 0.284)	− 0.074 (− 0.256, 0.109)
History of CVD event *(Reference: No)*			
*Yes*	0.048 (−0.126, 0.222)	0.026 (− 0.149, 0.202)	0.023 (− 0.113, 0.160)
Intercept			12.128 (11.775, 12.481)**
**Haemorrhagic stroke**			
**Variable**	**Univariate models** β (95% CI)	**Model 1** β (95% CI)	**Model 2** β (95% CI)
Age, *per year increase*	−0.004 (−0.012, 0.005)	−0.003 (− 0.012, 0.006)	0.001 (− 0.006, 0.009)
Sex (*Reference: Female)*			
*Male*	0.088 (−0.130, 0.305)	0.070 (− 0.154, 0.293)	0.042 (− 0.155, 0.238)
History of hypertension *(Reference: No)*			
*Yes*	0.315 (0.057, 0.687)*	0.316 (0.059, 0.692)*	−0.230 (− 0.560, 0.100)
History of diabetes *(Reference: No)*			
*Yes*	0.227 (−0.023, 0.477)	0.249 (−0.004, 0.502)	0.140 (− 0.088, 0.368)
History of atrial fibrillation *(Reference: No)*			
*Yes*	0.201 (−0.425, 0.828)	0.216 (−0.419, 0.851)	− 0.062 (− 0.647, 0.523)
History of CVD event *(Reference: No)*			
*Yes*	−0.063 (− 0.318, 0.192)	−0.070 (− 0.326, 0.187)	0.098 (− 0.138, 0.333)
Intercept			12.575 (11.935, 13.214)**

Parameter estimates from Generalized Linear models with gamma distribution and Log-link function. Model 1: Univariate models + adjusted for age and sex, Model 2: Multivariate model adjusting for age, sex, hypertension, diabetes, atrial fibrillation & previous CVD event and hospital attended. * < 0.05, ** < 0.01, ** < 0.001

## Discussion

In this study estimating the direct medical costs of major CVDs in Cameroon, IHD had the highest mean annual cost per patient followed by stroke and finally HHD. Combined estimates showed that stroke accounted for about two-thirds of total CVD costs in the study hospitals. A differential in mean cost per patient was observed between both hospitals, wherein with the exception of IHD, costs were mostly higher at the DGH. Overall, inpatient costs were two- to three-fold higher than outpatient costs. The determinants of cost varied by CVD, with diabetes predicting higher costs across all CVDs.

The mean annual cost of IHD in this study was XAF 1,395,200 (~US$ 2525), and this was substantially higher for acute compared to prevalent cases, with inpatient costs more than four-fold higher than outpatient costs. Given the absence of similar studies in Cameroon, and scant empirical data on the costs of coronary heart disease in Africa, we attempt to compare our findings with that elsewhere. A study in Hong Kong found that the mean direct medical cost of stable coronary heart disease was US$ 11,477 during the first year of diagnosis [21]. This is more than double the average annual cost of ‘acute’ IHD, referred to as first ever event in our study. Ribeiro et al. [22] estimated the mean annual cost of IHD to be R$ 2733 (~US$ 499.8) and R$ 6788 (~US$ 1241) in public and private sectors in Brazil, which are lower than our estimates. The differences in costs in these two studies compared to ours are likely due to differences in case mix and also the substantially higher cost of invasive therapeutic procedures carried out in the Hong Kong study, not otherwise done in our study. In addition, the Brazilian study [22] used diagnostic related groups, which has some dissimilarity with our micro-costing approach tracking patient-specific data. Underlying differences in costing approach and health systems could potentially explain some of the observed differences. The higher inpatient costs compared to outpatient costs for IHD found in our study is consistent with the literature, even though these estimates may vary depending on the duration of patient follow-up over which costs are tracked [8] and the costing perspective.

Our study showed an average annual cost of XAF 932,700 (~US$ 1688) for ischaemic stroke and XAF 815,400 (~US$ 1476) for haemorrhagic stroke. This is higher than estimates reported from Benin [23] but similar to a previous report in Cameroon [11]. Contrary to our study, haemorrhagic stroke has consistently been reported as having higher costs compared to ischaemic stroke [11, 23]. However, when we compared inpatient and outpatient costs, the inpatient costs for haemorrhagic stroke were higher than for ischaemic stroke, findings that are consistent with the literature [20, 24, 25]. In contrast to what obtained in the hospitals in our study, haemorrhagic stroke patients especially in affluent countries undergo neurosurgical procedures that substantially add to their costs compared to ischaemic stroke patients, which in part may explain some of the difference. Outpatient costs for ischaemic stroke patients were higher than for their haemorrhagic stroke counterparts, largely driven by physiotherapy. Ischaemic stroke patients have been shown to have comparatively better survival but slower recovery from neurological sequelae and thus likely require more rehabilitation, and resultant costs [26].

In our study, HHD had comparatively the lowest average annual cost per patient (XAF 384,300, ~US$ 695), and like the other CVDs, the cost was higher for ‘acute’ cases compared to prevalent, as well as higher inpatient costs compared to outpatient costs. This is the first study to estimate the cost of HHD in Cameroon and to our knowledge the first in Africa. In a cost-effectiveness analysis done alongside a clinical trial in the United States, Twiner et al. estimated the median annual cost of subclinical HHD at US$ 8015 in a sample of emergency department patients [27]. This was over five-fold higher than our study estimates. The inclusion of patient time and travel costs to their estimates, the selected nature of participants (trial) and differences in health services could in part explain the observed difference. In a Nigerian cohort of heart failure (predominantly due to hypertension) patients, the estimated cost of heart failure was US$ 2128 per patient per year [10], which is more than double our estimate. The higher costs observed in this study could be accounted for by the fact that in addition to including indirect costs, authors also included patient travel costs which together accounted for over 55% of total costs. In a recent review of cost-of-illness studies between 2004 and 2016, the cost of heart failure ranged between US$ 868 in South Korea to US$ 25,532 in Germany, and variations across estimates were largely due to difference in costing methodologies [28]. In our study, there was significant difference in healthcare costs between the hospitals in the study. This has been reported previously for stroke in Cameroon [24], and elsewhere [23, 29]. This is largely due to the differences in unit costs of medications and services between hospitals, the range of services, policies and treatment protocols.

Increasing age was associated with higher costs for ischaemic stroke in the age- and sex- adjusted models, and this is consistent with the literature [30]. With aging, people are likely to have had a number of comorbidities, visit more and stay longer in hospital requiring more healthcare use with ensuing costs compared to younger people. However, in the fully adjusted model, this age-cost relationship was attenuated. We did not find a significant relation between age and IHD or HHD costs. In a like manner, some authors have observed no relationship between age and CVD costs [8, 22]. Medical procedures (catheterization and angioplasty) were a major contributor to the higher IHD costs in our study. This is analogous to the results of Lee et al. in which newly diagnosed stable coronary artery disease patients who had invasive procedures incurred significantly higher (double) average healthcare costs compared to their counterparts who were only treated medically. Diabetes mellitus was a significant predictor of elevated costs in patients with IHD, ischaemic stroke and HHD. This is akin to findings in Brazil and Switzerland among IHD patients [8, 30]. In a study on the annual medical expenditures for hypertensive patients in the United States, healthcare costs were more than double in those with co-occurring diabetes [31]. Among stroke patients, Ng et al. found that average annual costs were higher with increasing number of comorbidities with dyslipidaemia, hypertension and diabetes being the most prevalent comorbidities [20]. Co- and multi-morbidity (as well as complications) have the tendency to increase healthcare costs for diseases, in part due to the added laboratory or imaging investigations as well as treatments warranted. These are also likely to contribute to prolonged hospital stay, which itself is positively correlated with healthcare costs. Presence of atrial fibrillation was associated with lower IHD costs in the age- and sex-adjusted models, but not in the fully adjusted model. In addition, having had a prior CVD event (that is, a prevalent case), was associated with lower costs in those with IHD. While the above observations may sound surprising, it is likely that patients with atrial fibrillation (already a high-risk group) did not undergo invasive cardiac procedures like catheterization due to potentially increased risk of mortality and anaesthetic complications and thus were treated mostly medically and had comparatively lower costs. In addition, it is possible that prevalent cases of IHD probably required less aggressive therapy and or diagnostic/imaging procedures compared to acute cases, thereby explaining the lower costs in these sub-group of patients.

Taken together, contrasting our findings with the existing literature requires some degree of caution. Dissimilarities in average healthcare cost estimates and drivers of costs are amongst others likely influenced by patient (clinical heterogeneity and case mix), study design (prevalence-based versus incidence-based studies, retrospective versus prospective), costing perspective (patient, provider or societal), approach (macro-costing versus micro-costing) health system (hospital management guidelines/protocols, available clinical/technical resources, unit costs for services) and time (impact of inflation over different years when studies were conducted) factors. As a result, interpretation of cost estimates should be done within the context of these heterogeneities.

### Limitations and strengths

This study is not without limitations. First, the retrospective collection of data could not exclude the likelihood of missing data, which could have influenced (under-estimation) the healthcare costs estimates. Second, this study included data only from two hospitals and thus our findings are most likely a representation of the situation at these hospitals. Given they were tertiary facilities, this has further implications for generalizability, as our cohort of patients are less likely to be representative of the entire country but rather of mostly those who could afford these facilities. Nevertheless, this is probably indicative of the care that should be available to all, as these hospitals are among the few that provide comparatively the most comprehensive care for CVDs in the country. Third, the absence of a control population, to compare and estimate the excess costs attributable to each CVD, and tease out the determinants of these excess costs. Fourth, our classification of acute versus prevalent could be seen as particular, given that we relied on reports of prior CVD event which were documented in their medical records. Inaccuracies here could have led to misclassification bias with potential impact on our cost estimates. Finally, we obtained unit costs for drugs not available in the hospital from external pharmacies. This may be anticipated to overestimate medication costs, particularly for non-generic forms of the drugs. In an attempt to mitigate this impact, substantial efforts were made to obtain unit costs for generic forms of the drug, and when this was not possible; the cheapest option for the non-generic form was used. While this is likely to reflect the situation of healthcare experienced by these patients, interpretation of our cost estimates should be done in the light of these circumstances.

Despite the above limitations, the relatively large sample size enabled us explore and to have a reasonable sense of the mean and distribution of healthcare costs for the CVDs in these hospitals. Secondly, we used robust statistical methods to investigate the determinants or factors that influenced the costs of CVDs. To the best of our knowledge, this is the first study in Cameroon and Africa to estimate the healthcare costs of IHD and hypertensive heart disease as well as investigate the drivers of these costs. Our results would thus serve as useful inputs for economic evaluations to comprehensively assess the cost-effectiveness of existing or potential CVD interventions in Africa.

### Implications for policy and research

Average annual costs of CVD per patient found in this study are about five to ten times higher than total health expenditure per capita (Int$ 122) in Cameroon [32]. This is substantial for a lower-middle income country where in addition, up to 37.5% (over 8.3 million) of the population live below the poverty line (less than XAF 936 – US$ 1.8 per day) [33]. In Cameroon, healthcare costs are borne mostly (almost 70%) by patients through out-of-pocket expenditure, and the rest mainly by the government and some from external funders. It is thus evident that a significant proportion of the population cannot meet the costs of care for CVDs, which are already posing a huge burden on the health system. However, the government via the ministry of health and national action strategies has since commenced efforts towards providing quality, accessible health services for all in its universal health coverage (UHC) agenda. This implies, in the very near future, there’s going to be some shift in the payer of these healthcare costs. Estimates of healthcare costs presented here would therefore be very vital for planning and priority setting as the government works towards setting up its UHC program. Potential considerations for health policy in the country;

- Standardize costs of healthcare services and medicines to avoid large differentials in costs across hospitals. This is particularly important as efforts at the Ministry of Health are currently underway to introduce UHC, which would hopefully provide leverage for the provision of quality and equitable healthcare towards attainment of SDG 3.8 by 2030 [34].
- Invest in cost-effective primary preventive interventions for risk factors at population-level [35] including legislation and taxation for tobacco, taxing unhealthy foods (high in salt, energy dense) and providing subsidies for healthy alternatives like fruits and vegetables, creating opportunities for active transport (cycling, walking paths) that will help improve risk profiles thereby decreasing incidence of CVDs and their resulting healthcare costs.

Directions for future research;

- Future cost-of-illness studies should be conducted across a wider and more representative range of health facilities in the national territory in order to better depict the national average costs of these CVDs and explore any regional differences in estimates and determinants of costs. Such studies should include control populations that will enable the ascertainment of excess costs attributable to CVDs.
- There is need for longitudinal or incidence-based cost of illness studies that will not only estimate the annual healthcare costs, but also in addition, track the lifetime costs of these CVDs. This would provide a more complete picture of the future costs of CVDs that would assist and guide decision-makers in resource allocation.

## Conclusions

This cost-of-illness study has provided a relatively comprehensive estimate of the economic burden, in terms of healthcare costs due to major CVDs in Cameroon. Comorbidities, particularly diabetes mellitus, and medical procedures were important drivers of elevated costs. These findings are important for health policy makers as it provides evidence of the contemporary economic burden of CVDs that would guide them in making informed decisions on priority setting and resource allocation for the prevention and control of CVD in Cameroon.

## Supplementary Information

**Additional file 1 Table S1** Distribution of study participants by town and region of residence according to hospital. **Table S2**. Comparison of the mean annual costs for ischaemic heart disease per patient between the two study hospitals (Doula General Hospital and Shisong Cardiac Centre) by cost components, 2013–2017, Cameroon. **Table S3.** Comparison of the mean annual costs for ischaemic stroke per patient between the two study hospitals (Doula General Hospital and Shisong Cardiac Centre) by cost components, 2013–2017, Cameroon. **Table S4**. Comparison of the mean annual costs for haemorrhagic stroke per patient between the two study hospitals (Doula General Hospital and Shisong Cardiac Centre) by cost components, 2013–2017, Cameroon. **Table S5.** Comparison of the mean annual costs for hypertensive heart disease per patient between the two study hospitals (Doula General Hospital and Shisong Cardiac Centre) by cost components, 2013–2017, Cameroon. **Table S6.** Comparison of mean annual costs for various CVDs per patient by region of residence, for patients at the Shisong Cardiac Centre, 2013–2017. **Table S7.** Comparison of mean annual costs for various CVDs per patient by region of residence, for patients at the Douala General Hospital, 2013–2017.

## Data Availability

All data generated or analysed during the current study are included in this published article and supporting files. Specific data on hospital cost profiles and resource use are not publicly available as they constitute sensitive information and was restricted by the study hospitals. The hospitals could be contacted for requests at http://www.hgdcam.com/ for DGH and P.O. Box 8, Kumbo, Northwest, Cameroon for SCC.

## Abbreviations

CVD: Cardiovascular disease
DGH: Douala general hospital
H-stroke: Haemorrhagic stroke
HHD: Hypertensive heart disease
ICD: International classification of diseases
ICU: Intensive care unit
IHD: Ischaemic heart disease
I-stroke: Ischaemic stroke
SCC: Shisong cardiac centre
USD: United States dollar
XAF: Central African franc
