# Supplementary material for: Estimation and determinants of direct medical costs of ischaemic heart disease, stroke and hypertensive heart disease: evidence from two major hospitals in Cameroon
Source: BMC Health Serv Res. 2021 Feb 12;21:140. doi: 10.1186/s12913-021-06146-4 (PMC7881453; doi:10.1186/s12913-021-06146-4)
Supplement: Supplementary file 1 — Additional file 1 Table S1 Distribution of study participants by town and region of residence according to hospital. Table S2. Comparison of the mean annual costs for ischaemic heart disease per patient between the two study hospitals (Doula General Hospital and Shisong Cardiac Centre) by cost components, 2013–2017, Cameroon. Table S3. Comparison of the mean annual costs for ischaemic stroke per patient between the two study hospitals (Doula General Hospital and Shisong Cardiac Centre) by cost components, 2013–2017, Cameroon. Table S4. Comparison of the mean annual costs for haemorrhagic stroke per patient between the two study hospitals (Doula General Hospital and Shisong Cardiac Centre) by cost components, 2013–2017, Cameroon. Table S5. Comparison of the mean annual costs for hypertensive heart disease per patient between the two study hospitals (Doula General Hospital and Shisong Cardiac Centre) by cost components, 2013–2017, Cameroon. Table S6. Comparison of mean annual costs for various CVDs per patient by region of residence, for patients at the Shisong Cardiac Centre, 2013–2017. Table S7. Comparison of mean annual costs for various CVDs per patient by region of residence, for patients at the Douala General Hospital, 2013–2017. [file 12913_2021_6146_MOESM1_ESM.docx]

**Supplementary file: Aminde et al. Estimation and determinants of direct medical costs of ischaemic heart disease, stroke and hypertensive heart disease: evidence from Cameroon.**

**Table 1: Distribution of study participants by town and region of residence according to hospital**

| **Town of residence** | **SCC**  n = 251 | **DGH**  n = 599 | **Total** |
| --- | --- | --- | --- |
| Same town as hospital | 45 (17.9) | 421 (70.4) | 466 (54.9) |
| Out of study hospital town | 206 (82.1) | 177 (29.6) | 383 (45.1) |
| **Region of residence** |  |  |  |
| Far-North | 2 (0.8) | 5 (0.8) | 7 (0.8) |
| North | 2 (0.8) | 5 (0.8) | 7 (0.8) |
| Adamawa | 8 (3.2) | 6 (1.0) | 14 (1.6) |
| Centre | 22 (8.8) | 8 (1.3) | 30 (3.5) |
| Littoral | 22 (8.8) | 473 (79.1) | 495 (58.3) |
| East | 3 (1.2) | 5 (0.8) | 8 (0.9) |
| West | 23 (9.2) | 46 (7.7) | 69 (8.1) |
| North-West | 129 (51.4) | 12 (2.0) | 141 (16.6) |
| South-West | 34 (13.5) | 29 (4.8) | 63 (7.4) |
| South | 3 (1.2) | 6 (1.0) | 9 (1.1) |
| Neighbouring countries* | 3 (1.2) | 3 (0.2) | 6 (0.7) |

DGH = Douala General Hospital, SCC = Shisong Cardiac Centre, SD = standard deviation,

*****Nigeria = 1, Chad = 4, Togo = 1

**Table 2: Comparison of the mean annual costs for ischaemic heart disease per patient between the two study hospitals (Doula General Hospital and Shisong Cardiac Centre) by cost components, 2013 – 2017, Cameroon**

|  | **DGH**  n = 40 | **SCC**  n = 52 |
| --- | --- | --- |
| **Consultation** |  |  |
| Mean ± SD | 9,800 ± 6,300 | 3,500 ± 500 |
| Median (25^th^, 75^th^ percentile) | 7,000 (7,000 – 7,000) | 3,500 (3,500 – 3,500) |
| **Laboratory** |  |  |
| Mean ± SD | 142,800 ± 62,100 | 60,500 ± 13,900 |
| Median (25^th^, 75^th^ percentile) | 132,600 (102,500 – 173,800) | 63,400 (63,400 – 63,400) |
| **Imaging & procedures** |  |  |
| Mean ± SD | 97,500 ± 64,500 | 1,821,600 ± 1,399,900 |
| Median (25^th^, 75^th^ percentile) | 61,300 (50,800 – 130,300) | 695,500 (685,000 – 3,365,000) |
| **Medication** |  |  |
| Mean ± SD | 165,000 ± 168,000 | 206,100±208,200 |
| Median (25^th^, 75^th^ percentile) | 121,900 (54,200 – 210,500) | 133,300 (59,600 – 285,700) |
| **Hospitalization** |  |  |
| Mean ± SD | 67,400 ± 57,400 | 5,500 ± 3,400 |
| Median (25^th^, 75^th^ percentile) | 56,800 (42,600 – 70,400) | 5,000 (3,000 – 6,000) |
| **Total** |  |  |
| Mean ± SD | 482,400 ± 231,800 | 2,097,300 ± 1,461,900 |
| Median (25^th^, 75^th^ percentile) | 437,300 (315,100 – 584,300) | 1,163,000 (825,200 – 3,557,400) |

DGH = Douala General Hospital, SCC = Shisong Cardiac Centre, SD = standard deviation; Mean cost for imaging & procedures was significantly higher in SCC than DGH (p<0.001), no difference for medication costs between hospitals (p = 0.312), mean total costs was higher in SCC (p<0.001), the rest were higher in DGH than SCC (all p <0.01). Costs are reported in Central African Francs (XAF) and rounded to nearest hundred.

**Table 3: Comparison of the mean annual costs for ischaemic stroke per patient between the two study hospitals (Doula General Hospital and Shisong Cardiac Centre) by cost components, 2013 – 2017, Cameroon**

|  | **DGH**  n = 272 | **SCC**  n = 45 |
| --- | --- | --- |
| **Consultation** |  |  |
| Mean ± SD | 15,400 ± 9,600 | 3,500 ± 500 |
| Median (25^th^, 75^th^ percentile) | 14,000 (7,000 – 21,000) | 3,500 (3,500 – 3,500) |
| **Laboratory** |  |  |
| Mean ± SD | 124,200 ± 70,500 | 21,400 ± 8,400 |
| Median (25^th^, 75^th^ percentile) | 113,600 (72,700 – 150,100) | 20,200 (16,400 – 25,500) |
| **Imaging & procedures** |  |  |
| Mean ± SD | 211,800 ± 107,400 | 31,500 ± 14,900 |
| Median (25^th^, 75^th^ percentile) | 191,000 (125,800 – 273,400) | 30,000 (30,000 – 30,000) |
| **Medication** |  |  |
| Mean ± SD | 364,600 ± 240,700 | 144,300 ± 104,400 |
| Median (25^th^, 75^th^ percentile) | 320,000 (197,100 – 478,400) | 121,600 (74,600 – 190,500) |
| **Hospitalization** |  |  |
| Mean ± SD | 201,000 ± 133,100 | 8,000 ± 4,000 |
| Median (25^th^, 75^th^ percentile) | 133,100 (71,000 – 284,300) | 7,000 (5,000 – 10,800) |
| **Physiotherapy** |  |  |
| Mean ± SD | 638,500 ± 492,900 | 20,000 ± 15,000 |
| Median (25^th^, 75^th^ percentile) | 494,000 (161,500 – 1,035,500) | 15,000 (15,000 – 30,000) |
| **Total** |  |  |
| Mean ± SD | 1,054,000 ± 653,200 | 199,500 ± 112,800 |
| Median (25^th^, 75^th^ percentile) | 899,700 (630,900 – 1,324,400) | 178,700 (111,000 – 255,300) |

DGH = Douala General Hospital, SCC = Shisong Cardiac Centre, SD = standard deviation; Mean costs were higher in DGH compared to SCC (all p <0.01, independent samples t-test). Costs are reported in Central African Francs (XAF) and rounded to nearest hundred.

**Table 4: Comparison of the mean annual costs for haemorrhagic stroke per patient between the two study hospitals (Doula General Hospital and Shisong Cardiac Centre) by cost components, 2013 – 2017, Cameroon**

|  | **DGH**  n = 159 | **SCC**  n = 34 |
| --- | --- | --- |
| **Consultation** |  |  |
| Mean ± SD | 11,900 ± 7,200 | 3,500 ± 500 |
| Median (25^th^, 75^th^ percentile) | 7,000 (7,000 – 14,000) | 3,500 (3,000 – 3,500) |
| **Laboratory** |  |  |
| Mean ± SD | 116,300 ± 79,900 | 23,000 ± 12,000 |
| Median (25^th^, 75^th^ percentile) | 97,400 (69,000 – 137,400) | 22,000 (19,200 – 25,800) |
| **Imaging & procedures** |  |  |
| Mean ± SD | 217,300 ± 335,200 | 33,000 ± 15,000 |
| Median (25^th^, 75^th^ percentile) | 175,000 (90,000 – 225,800) | 30,000 (30,000 – 30,000) |
| **Medication** |  |  |
| Mean ± SD | 313,500 ± 237,000 | 197,000 ± 95,000 |
| Median (25^th^, 75^th^ percentile) | 294,500 (135,600 – 425,400) | 185,400 (122,800 – 263,300) |
| **Hospitalization** |  |  |
| Mean ± SD | 300,700 ± 282,800 | 12,100 ± 4,500 |
| Median (25^th^, 75^th^ percentile) | 285,200 (80,800 – 411,800) | 13,000 (8,000 – 15,300) |
| **Physiotherapy** |  |  |
| Mean ± SD | 628,200 ± 289,500 | 38,800 ± 28,600 |
| Median (25^th^, 75^th^ percentile) | 641,300 (389,500 – 755,300) | 30,000 (22,000 – 45,000) |
| **Total** |  |  |
| Mean ± SD | 931,900 ± 724,800 | 270,700 ± 100,900 |
| Median (25^th^, 75^th^ percentile) | 797,900 (372,200 – 1,192,100) | 275,200 (181,000 – 338,100) |

DGH = Douala General Hospital, SCC = Shisong Cardiac Centre, SD = standard deviation; Mean costs were higher in DGH compared to SCC (all p <0.01, independent samples t-test). Costs are reported in Central African Francs (XAF) and rounded to nearest hundred.

**Table 5: Comparison of the mean annual costs for hypertensive heart disease per patient between the two study hospitals (Doula General Hospital and Shisong Cardiac Centre) by cost components, 2013 – 2017, Cameroon**

|  | **DGH**  n = 128 | **SCC**  n = 120 |
| --- | --- | --- |
| **Consultation** |  |  |
| Mean ± SD | 11,100 ± 8,900 | 3,500 ± 1,000 |
| Median (25^th^, 75^th^ percentile) | 7,000 (7,000 – 7,000) | 3,500 (3,500 – 3,500) |
| **Laboratory** |  |  |
| Mean ± SD | 176,600 ± 105,400 | 20,300 ± 11,200 |
| Median (25^th^, 75^th^ percentile) | 147,400 (105,000 – 224,800) | 19,300 (12,600 – 24,300) |
| **Imaging & procedures** |  |  |
| Mean ± SD | 103,500 ± 158,300 | 35,800 ± 16,200 |
| Median (25^th^, 75^th^ percentile) | 61,300 (50,800 – 122,400) | 30,000 (30,000 – 30,000) |
| **Medication** |  |  |
| Mean ± SD | 199,300 ± 140,300 | 130,800 ± 75,700 |
| Median (25^th^, 75^th^ percentile) | 194,000 (82,700 – 271,000) | 122,100 (73,200 – 173,600) |
| **Hospitalization** |  |  |
| Mean ± SD | 79,700 ± 69,500 | 7,900 ± 4,600 |
| Median (25^th^, 75^th^ percentile) | 71,000 (42,600 – 99,400) | 7,000 (5,000 – 10,000) |
| **Total** |  |  |
| Mean ± SD | 567,200 ± 315,600 | 189,200 ± 82,000 |
| Median (25^th^, 75^th^ percentile) | 508,600 (370,400 – 687,200) | 182,500 (128,000 – 239,000) |

DGH = Douala General Hospital, SCC = Shisong Cardiac Centre, SD = standard deviation; Mean costs were higher in DGH compared to SCC (all p <0.001, independent samples t-test). Costs are reported in Central African Francs (XAF) and rounded to nearest hundred.

**Table 6: Comparison of mean annual costs for various CVDs per patient by region of residence, for patients at the Shisong Cardiac Centre, 2013 – 2017.**

| **Shisong Cardiac Centre (SCC)** | **Same region (North-West)** | **Other region** | **p-value** |
| --- | --- | --- | --- |
| Ischaemic heart disease | 1,404,800 ± 1,322,100 | 2,242,300 ± 1,462,200 | 0.119 |
| Ischaemic stroke | 172,300 ± 106,300 | 213,100 ± 115,200 | 0.258 |
| Haemorrhagic stroke | 274,300 ± 104,900 | 261,800 ± 95,300 | 0.748 |
| Hypertensive heart disease | 188,300 ± 86,400 | 190,300 ± 77,000 | 0.895 |

Estimates are mean ± standard deviation (SD) and costs are reported in Central African Francs (XAF)

**Table 7: Comparison of mean annual costs for various CVDs per patient by region of residence, for patients at the Douala General Hospital, 2013 – 2017.**

| **Douala General Hospital (DGH)** | **Same region (Littoral)** | **Other region** | **p-value** |
| --- | --- | --- | --- |
| Ischaemic heart disease | 494,500 ± 240,200 | 374,100 ± 90,900 | 0.331 |
| Ischaemic stroke | 1,044,600 ± 648,600 | 1,084,500 ± 672,000 | 0.670 |
| Haemorrhagic stroke | 908,300 ± 640,900 | 1,012,300 ± 965,000 | 0.450 |
| Hypertensive heart disease | 577,500 ± 332,500 | 517,600 ± 215,600 | 0.420 |

Estimates are mean ± standard deviation (SD) and costs are reported in Central African Francs (XAF)
